# Morphogenetic Studies of the *Drosophila* DA1 Ventral Olfactory Projection Neuron

**DOI:** 10.1371/journal.pone.0155384

**Published:** 2016-05-10

**Authors:** Hung-Chang Shen, Jia-Yi Wei, Sao-Yu Chu, Pei-Chi Chung, Tsai-Chi Hsu, Hung-Hsiang Yu

**Affiliations:** 1 Institute of Cellular and Organismic Biology, Academia Sinica, Taipei, Taiwan; 2 Graduate Institute of Life Sciences, National Defense Medical Center, Taipei, Taiwan; Tohoku University, JAPAN

## Abstract

In the *Drosophila* olfactory system, odorant information is sensed by olfactory sensory neurons and relayed from the primary olfactory center, the antennal lobe (AL), to higher olfactory centers via olfactory projection neurons (PNs). A major portion of the AL is constituted with dendrites of four groups of PNs, anterodorsal PNs (adPNs), lateral PNs (lPNs), lateroventral PNs (lvPNs) and ventral PNs (vPNs). Previous studies have been focused on the development and function of adPNs and lPNs, while the investigation on those of lvPNs and vPNs received less attention. Here, we study the molecular and cellular mechanisms underlying the morphogenesis of a putative male-pheromone responding vPN, the DA1 vPN. Using an intersection strategy to remove background neurons labeled within a DA1 vPN-containing GAL4 line, we depicted morphological changes of the DA1 vPN that occurs at the pupal stage. We then conducted a pilot screen using RNA interference knock-down approach to identify cell surface molecules, including Down syndrome cell adhesion molecule 1 and Semaphorin-1a, that might play essential roles for the DA1 vPN morphogenesis. Taken together, by revealing molecular and cellular basis of the DA1 vPN morphogenesis, we should provide insights into future comprehension of how vPNs are assembled into the olfactory neural circuitry.

## Introduction

Ensembles of neurons are linked into complex neural circuits in the nervous system in which animals use them to process environmental information, e.g., light, sound and odors, etc., for survival and offspring reproduction. A series of steps must occur in the formation of the neural circuitry: stringent regulation of generation and survival of the number of neurons, cell fate specification after the birth of neurons, accurate navigation of neuronal axons and dendrites to their targets, appropriate patterns of axonal branches and dendritic arborizations within neurons and correct connections among neurons for assembling into functional neural circuits [1]. Comprehensive identification of genes and molecules that regulate each cellular step described above should provide insights into how the complex neural circuits are formed in the brain throughout the animal kingdom.

In the *Drosophila* olfactory system, odors are detected by ~50 classes of ~1,300 olfactory sensory neurons (OSNs) in the antennae and maxillary palps [2]. Odorant inputs are then delivered by OSN axons to the primary olfactory center, the antennal lobe (AL), where OSN axons make connections with neurites of ~250 projection neurons (PNs) and local interneurons (LNs) [2, 3]. Odorant signals are modulated by LNs, and then relayed by PNs to higher olfactory centers, e.g., mushroom body and lateral horn (LH), for further decoding of the olfactory information [2]. These PNs and LNs are derived from at least five neural stem cells; the derived neurons include three clusters of PNs (anterodorsal PNs (adPNs in the ALad1 lineage), ventral PNs (vPNs in the ALv1 lineage), and lateroventral PNs (in the ALlv1 lineage)), a set of ventral LNs (in the ALv2 lineage), and a lateral population of mixed PNs and LNs (in the ALl1 lineage) [3]. Of these four PN groups, adPNs and PNs from the ALl1 lineage (lPNs) have been extensively explored at the molecular, cellular and functional levels, while the investigation of those of vPNs and lvPNs received less attention [4]. Revealing the molecular and cellular mechanisms underlying the morphogenesis of vPNs and lvPNs will advance our understanding of how multiple populations of neurons are integrated into the functional olfactory system during development.

Here, we use the DA1 vPN, a neuron anatomically receiving the input from a class of male-pheromone responding OSNs (Or67d OSNs), as an entry to study the molecular and cellular mechanisms underlying the morphogenesis of vPNs [5, 6]. We identified R95B09-GAL4 which labels the DA1 vPN within the AL together with background neurons outside of the AL from the JFRC GAL4 collection [7]. We then obtained a pure DA1 vPN labeling pattern by an intersection strategy to remove background neurons from R95B09-GAL4, allowing us not only to depict DA1 vPN morphogenesis but also to utilize it for the DA1 vPN phenotypic analysis in loss-of-function study. Finally, to prove in principle that R95B09-GAL4 is a good reagent for investigating the DA1 vPN development at the molecular level, we conducted a pilot RNA interference (RNAi) knock-down screen and identified cell surface molecules, including Down syndrome cell adhesion molecule 1 (Dscam1) and Semaphorin-1a (Sema-1a), that might participate in the DA1 vPN morphogenesis [8]. Taken together, using R95B09-GAL4 we revealed molecular and cellular basis of the DA1 vPN morphogenesis, which sets up a foundation for future comprehensive understanding of DA1 vPN-mediated biological processes and neural circuitry assembly in the olfactory system.

## Results

### Exploration of the use of R95B09-GAL4 as a tool for studying DA1 vPN morphogenesis

**Fig 1:**
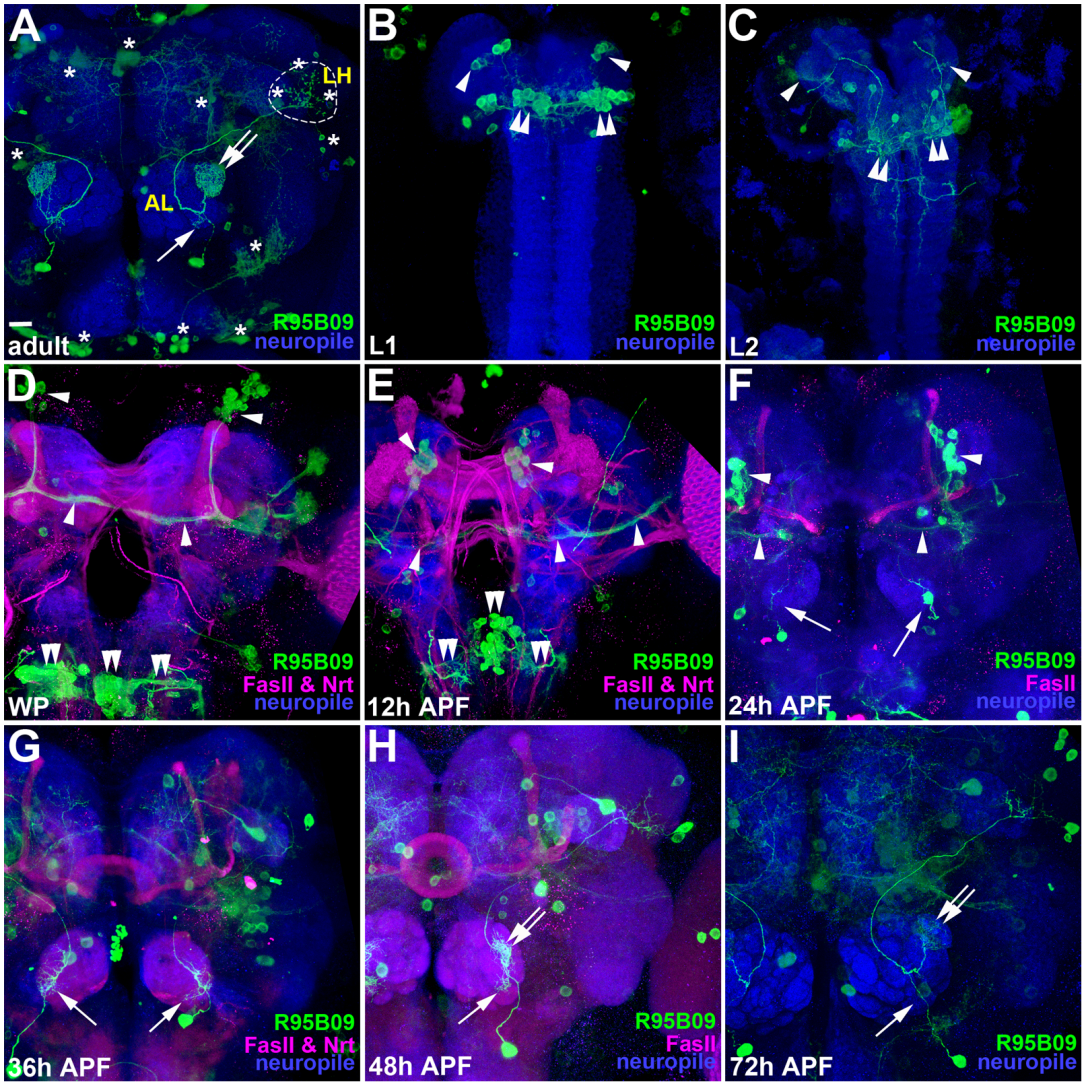
Expression pattern of R95B09-GAL4 during development. (A-H) The expression pattern of R95B09-GAL4 during development was revealed by using it to drive the expression of the mCD8::GFP reporter (green). The genotypes of Figs 1–3 and S1 Fig are summarized in S2 Table. Brain neuropiles shown in blue were stained with antibodies against Bruchpilot (Brp; A-C and I) or DN-cadherin (D-H). Staining against Fasciclin II (FasII) and/or Neurotactin (Nrt) shown in magenta was performed to reveal landmarks for comparison of the relative position of the developing DA1 vPN in D-H. Ventral and dorsal dendrites of the DA1 vPN (arrow and double-arrow in A) were clearly observed in the AL, while the axonal pattern was partially blocked by background neurons in the LH (three asterisks within dash-circle in A). The developing DA1 vPN was detected after puparium formation (APF; arrows and double-arrows in D-I). Background neurons were indicated with asterisks in the adult brain (A) and they were also seen in the brains of different developmental stages with a transient and yet complex pattern (B-I). Two groups of background neurons were seen from early larval to pupal stages (B-I): the first group is dorsal neurons that project their neurites ventrally to the middle of the central brain and then bifurcate their neurites medially and laterally toward the midline and optic lobes (arrowheads in Fig 1B–1F); the other one is a group of neurons in which their cell bodies locate between the central brain and the ventral nerve cord and their neuritis project medially to the midline (double-arrowheads in Fig 1B–1E). First instar larval (L1), second instar larval (L2) and white pupal (WP). Scale bar: 10 μm for A-I, except for D as 14 μm.

To identify useful GAL4 lines that label vPNs, we sought to search for the Janelia GAL4 collection by viewing through imaging files in the website of Bloomington *Drosophila* stock center (http://flystocks.bio.indiana.edu/Browse/gal4/gal4_Janelia.php) that portray expression patterns of many available GAL4 lines. Fortunately, we identified R95B09- GAL4 (Bloomington stock number (BL) 47276) which labels a single DA1 vPN (arrow and double-arrow in Fig 1A) together with background neurons in brain regions outside of the AL (asterisks in Fig 1A) [7]. For this DA1 vPN, the dense dendritic innervation was clearly seen in the DA1 glomerulus at the AL (double-arrow in Fig 1A), while some loose dendritic arborizations were also detected in the ventral AL (arrow in the Fig 1A). In contrast, axonal elaboration pattern of the DA1 vPN cannot be unambiguously identified in the LH due to the presence of background neurons in the proximity of the same LH region (dash-circle for DA1 vPN axonal branches and three asterisks in the dash-circle for background neurons in Fig 1A). We sought to identify whether R95B09-GAL4 labels the DA1 vPN throughout morphogenesis since GAL4 lines may label neurons in a transient and yet complex pattern over the course of development that makes them unsuitable for examining the effects of genetic perturbation on the development of neurons of interest (e.g., a group of dorsal neurons that project their neurites ventrally to the middle of the central brain and bifurcate their neurites medially and laterally to the midline and optic lobes can be seen from early larval to early pupal stages (pointed by arrowheads in Fig 1B–1F), but they gradually diminished after mid-late pupal stage (Fig 1G–1I)). We then expressed mCD8::GFP under control of R95B09-GAL4 and examined the expression pattern of this GAL4 from the early larval stage onwards through adulthood (Fig 1A–1I). Besides those dorsal neurons, neuronal expression was also detected in a group of medially projected neurons whose cell bodies locate between the central brain and the ventral nerve cord in R95B09-GAL4 started from the early larval stage (double-arrowheads in Fig 1B–1E). In contrast, neuronal expression in the AL was not apparent in R95B09-GAL4 until after puparium formation (APF; arrow in Fig 1F–1I). Interestingly, a single neuron (most likely the DA1 vPN) innervating the AL was clearly observed in R95B09-GAL4 from 24-hour APF to the adult (Fig 1A and 1F–1I). This result suggested that R95B09-GAL4 may be ideally utilized for perturbing gene function and then examining the causal phenotype in the DA1 vPN.

**Fig 2:**
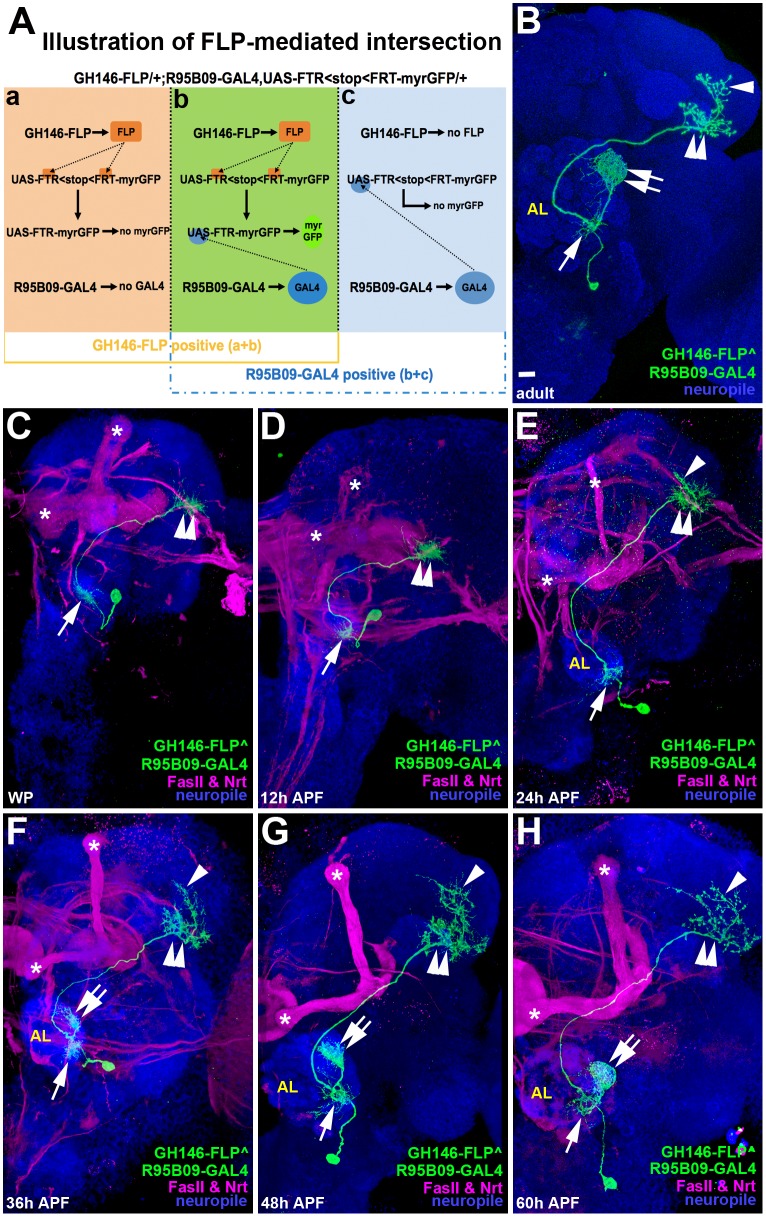
Use of an intersection strategy to visualize the developing DA1 vPN. (A) A schematic illustration of how to obtain a pure DA1 vPN expression pattern by intersecting the expression of a membrane bound GFP reporter, myristoyl-anchored GFP (myr-GFP) reporter, with R95B09-GAL4 and GH146-FLP. All neurons contain the genetic components of *GH146-FLP*, *R95B09-GAL4* and *UAS-FRT<stop<FRT-myr-GFP* in the genome. However, the expression of myr-GFP is initially blocked by the presence of a FRT<stop<FRT cassette. GH146-FLP is expressed to remove this stop cassette in neurons of panels a and b but not in those of panel c. On the other hand, R95B09-GAL4 is expressed in neurons of panel b and c but not in those of panel a. Therefore, myr-GFP can be only expressed in neurons of panel b in which GH146-FLP and R95B09-GAL4 are co-expressed. (B) A flattened confocal image revealed the pure DA1 vPN expression pattern that was obtained by intersecting R95B09-GAL4 and GH146-FLP. Dense and loose dendritic innervations in the DA1 glomerulus and the ventral portion of the AL were pointed by double-arrow and arrow, respectively. Characteristic dorsal and ventral axonal branches were indicated by arrowhead and double-arrowhead, respectively. (C-H) The morphogenesis of the developing DA1 vPN can be observed in confocal images taken from the brains of *Drosophila* in which R95B09-GAL4 was intersected with GH146-FLP; brains were processed at the following stages: white pupa (WP) (C), 12-hour (D), 24-hour (E), 36-hour (F), 48-hour (G), and 60-hour (H) after puparium formation (APF). In WP, axons and dendrites of the DA1 vPN were present at the ventral portions of the developing LH and AL, respectively. The characteristic dorsal axonal branches of the DA1 vPN started to appear around 24-hour APF (arrowhead in E) and became increasingly prominent afterward (arrowheads in F-H). Ventral axonal braches were also observed to elaborate after 24-hour APF (double-arrowheads in C-H). In contrast, the DA1 vPN dendrites remained at the ventrolateral developing AL prior to 36-hour APF (arrows in C-H) and started to elaborate dorsally in the developing AL at around 36-hour AFP (double-arrow in F). Gradually, the DA1 vPN dendrites accumulated at the dorsolateral part of the developing AL, with a resulting reduction of dendrites at the ventrolateral developing AL from 36-hour to 60-hour APF (double-arrows in F-H). The morphology of the developing DA1 vPN at 60-hour APF (H) was quite similar to that of the adult DA1 vPN (B). Brain neuropiles were stained with antibodies against Brp (A and B) or DN-cadherin (C-H) (shown in blue). Staining against FasII and Nrt (shown in magenta) was performed to reveal landmarks for comparison of the relative position of the developing DA1 vPN in C-H. Scale bar: 10 μm.

Prior to using R95B09-GAL4 as a manipulation tool, we wondered whether the background neurons labeled by R95B09-GAL4 could be removed through an intersection strategy, in which flippase (FLP) was used to remove a FLP-recognition-target (FRT)>stop>FRT cassette that blocks the expression of reporters (Fig 2A for the schematic illustration of this intersection strategy) [9]. Notably, a pure DA1 vPN labeling pattern was obtained by intersecting R95B09-GAL4 with a PN-expressing FLP line, GH146-FLP (Fig 2B), in which not only dendritic innervations were clearly visualized in the AL (arrow and double-arrow for the ventral AL and the DA1 glomerulus in Fig 2B) but also axonal arborizations were apparently detected as dorsal and ventral groups of axonal branches in the LH (arrowhead and double-arrowhead in Fig 2B) [10]. This clean DA1-vPN labeling pattern provides single cell resolution that can be used as a tool to directly characterize the morphological progression of the developing DA1 vPN and to examine the effects of manipulating gene expression in the DA1 vPN morphogenesis (both of them will be described below).

### Depiction of DA1 vPN morphogenesis by intersecting R95B09-GAL4 with GH146-FLP

Since the presence of background neurons in R95B09-GAL4 hinders a clear depiction of how the DA1 vPN develops, we then used the above FLP-out strategy with the intersection of GH146-FLP and R95B09-GAL4 to examine the morphogenesis of the DA1 vPN (Fig 2C–2H). Axonal branches were initially observed to occupy at the ventral part of the developing LH (double-arrowheads in the Fig 2C and 2D) and these ventral axonal branches became more elaborated after 24-hour APF (double-arrowheads in Fig 2B and 2E–2H). Intriguingly, characteristic dorsal axonal branches did not appear until 24-hour APF (Fig 2C–2E; arrowhead pointed at dorsal axonal branches in Fig 2E) and subsequently became more prominent (arrowheads in Fig 2B and 2F–2H). On the other hand, dendrites of the DA1 vPN were initially seen to distribute ventrally in the developing AL within the first 24 hours APF (arrows in Fig 2C–2E). The typical dorsal dendrites of the DA1 vPN were not visible in the developing AL until around 36-hour APF (double-arrow in Fig 2F). Dorsal dendrites of the DA1 vPN gradually accumulated and became denser from 36-hour to 60-hour APF (double-arrows in Fig 2F–2H), which eventually led to restriction of most of the dendrites to the DA1 glomerulus at the adult stage (double-arrow in Fig 2B). In contrast, the ventral dendrites of the DA1 vPN were gradually diminished during the pupal stage, becoming minimal processes by the adult stage (arrows in Fig 2B and 2F–2H). We should note that animals homozygous for GH146-FLP, R95B09-GAL4, and flip-out-cassette GFP reporter occasionally contained visible developing DA1 vPN between the white pupa stage (WP) and 12-hour APF (~10% and ~30%, respectively; n>20; Fig 2C and 2D), while the developing DA1 vPN could be detected in nearly 100% of heterozygous animals of the same genotype from 24-hour APF to the adulthood (n>20; Fig 2B and 2E–2H). Taken together, the above results suggested that the GAL4 of R95B09-GAL4 should be stably expressed in the DA1 vPN after 24-hour APF during DA1 vPN morphogenesis, which makes R95B09-GAL4 an ideal tool to examine the effect of perturbing gene expression on the development of the DA1 vPN.

### Identification of cell surface molecules that participate in DA1 vPN morphogenesis

**Fig 3:**
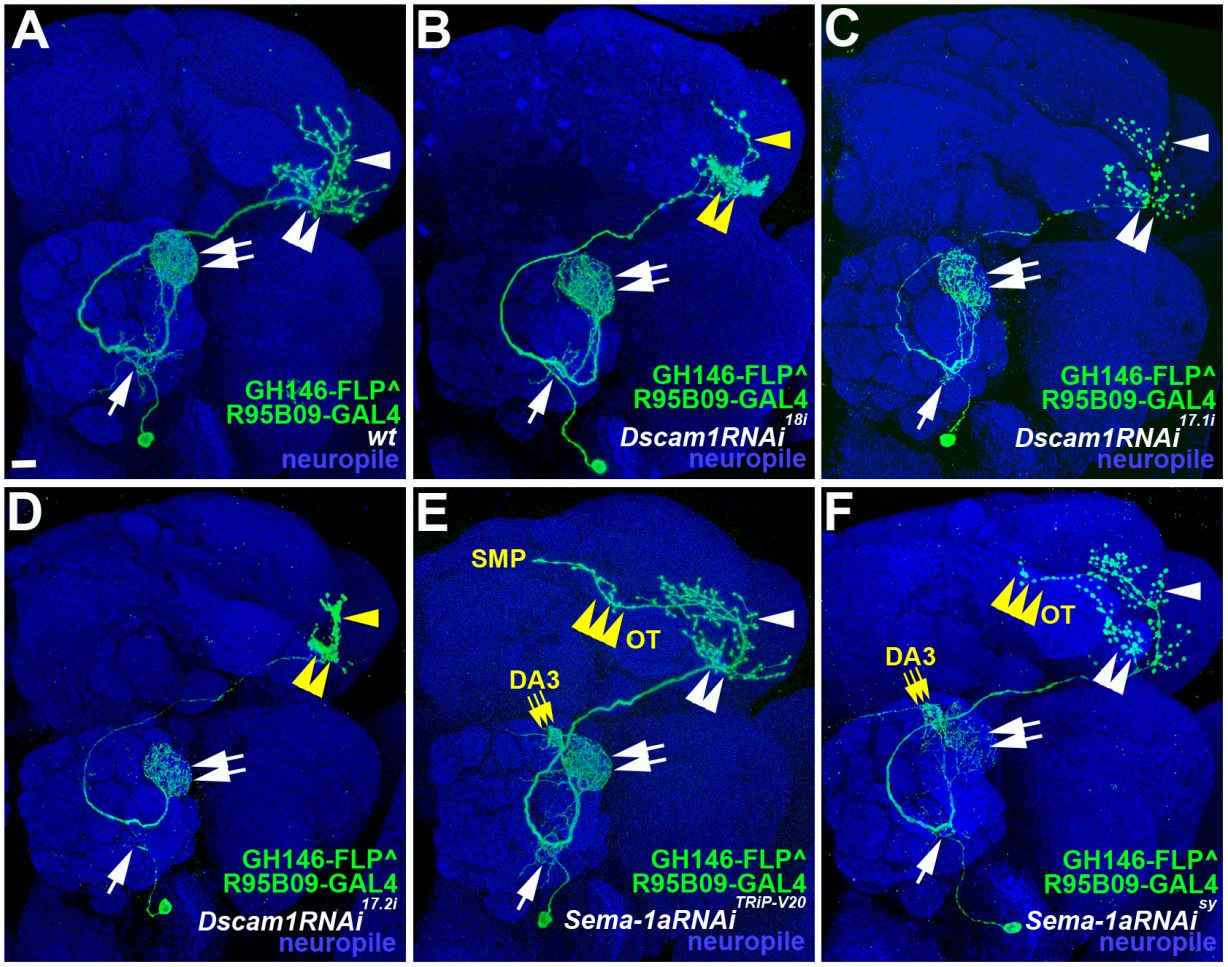
Using R95B09-GAL4 as a tool to identify *Dscam1* and *Sema-1a* essential for the DA1 vPN morphogenesis. Images of DA1 vPN phenotypes with gene perturbation were shown in green: wild-type (A), RNAi knock-down of *Dscam1* (B-D) and *Sema-1a* (E and F). Brain neuropiles were stained with an antibody against Brp (shown in blue in A-F). Dense and loose dendritic innervations in the DA1 glomerulus and the ventral portion of the AL were pointed by double-arrows and arrows in A-D. Dorsal and ventral axonal branches were indicated by arrowheads and double-arrowheads in A, C, E and F. (B and D) In *Dscam1RNAi*^*18i*^ and *Dscam1RNAi*^*17*.*2i*^ knock-down animals, ventral axons of the DA1 vPN appeared to aggregate in the LH (yellow double-arrowheads) that might result in the failure of branching out dorsal axons (yellow arrowheads). (C) In *Dscam1RNAi*^*17*.*1i*^ knock-down animals, no axonal phenotype was observed as compared to the *Dscam1RNAi*^*18i*^ and *Dscam1RNAi*^*17*.*2i*^ knock-down animals (double-arrowhead and arrowhead). (E and F) Axonal and dendritic phenotypes were observed in the DA1 vPNs expressing two independent *Sema-1a* RNAi transgenes, *Sema-1aRNAi*^*TRiP-V20*^ (BL34320) and *Sema-1aRNAi*^*sy*^ (home-made in the current study): mis-projected axons (yellow triple-arrowhead) were seen to channel through the optic tubercle (OT) to reach the superior medial protocerebrum (SMP); dendrites were also found to mis-target to the DA3 glomerulus (yellow triple-arrow). Scale bar: 10 μm.

Since cell surface molecules have been shown to participate in axonal and dendritic development [11, 12], we next sought to take RNAi knock-down approach to inhibit the expression of cell surface molecules that may affect the morphogenesis of the DA1 vPN (Fig 3A for a wild-type DA1 vPN). Just to prove in principle that R95B09-GAL4 can be served as an ideal tool for identifying essential cell surface molecules in the DA1 vPN morphogenesis, we utilized R95B09-GAL4 to drive the expression of a *Dscam1*-RNAi transgene, *Dscam1RNAi*^*18i*^, which potentially knocks down all Dscam1 isoforms by specifically targeting the common exon 18 of *Dscam1* that has previously shown to affect the axonal and dendritic development of vPNs [13]. Notably, a highly penetrant phenotype was observed in the axonal morphogenesis of the *Dscam1-*deficient DA1 vPN, in which the axons were unable to elaborate at the LH, resulting in crumpling up ventral axonal branches and failing to sprout out most of dorsal axonal branches (yellow double-arrowhead and yellow arrowhead in Fig 3B; phenotypic percentage of all RNAi knock-down results can be seen in S1A Fig). We then used two additional *Dscam1-*RNAi transgenic lines to confirm the finding of *Dscam1RNAi*^*18i*^-derived axonal defects: *Dscam1RNAi*^*17*.*1i*^ and *Dscam1RNAi*^*17*.*2i*^ can specifically silence dendritic- and axonal-expressed Dscam1 isoforms by targeting exons 17.1 and 17.2 of *Dscam1*, respectively [13]. Interestingly, the axonal phenotypes of crumpling up ventral axonal branches and failing to sprout out most of dorsal axonal branches were recapitulated by the expression of *Dscam1RNAi*^*17*.*2i*^ but not *Dscam1RNAi*^*17*.*1i*^ (double-arrowhead and arrowhead in Fig 3C for *Dscam1RNAi*^*17*.*1i*^ knock-down samples; yellow double- arrowhead and yellow arrowhead in Fig 3D for *Dscam1RNAi*^*17*.*2i*^ knock-down samples). We should note that no dendritic phenotype was observed by the expression of any of three *Dscam1*-RNAi transgenes (double-arrows and arrows in Fig 3B–3D). Taken together, these *Dscam1*-RNAi knock-down results suggested that R95B09-GAL4 is indeed an excellent reagent for the molecular study of the DA1 vPN development.

We then performed a pilot screen using R95B09-GAL4 to express RNAi transgenes carried by fly stocks available in our hand at the time to knock down the expression of 34 genes (all of them are available at the Bloomington *Drosophila* stock center; S1 Table): we found that loss-of-function (LOF) of *Sema-1a* displayed a highly penetrant phenotype on the axonal and dendritic morphogenesis of the DA1 vPN. By crossing the R95B09-GAL4 line with the *UAS-Sema-1aRNAi*^*TRiP-V20*^ transgenic line (which expresses *Sema-1a-*short hairpin RNA (shRNA) using the Valium 20 backbone vector [8]; BL34320), we observed that their progeny exhibited both axonal and dendritic phenotypes: (1) axons mis-projected away from the LH to the lateral edge of the superior medial protocerebrum (SMP) via the dorsal bottle neck of the optic tubercle (OT; yellow triple-arrowhead in Fig 3E); (2) dendrites were no longer limited to the DA1 glomerulus and mis-targeted into the DA3 glomerulus (yellow triple-arrow in Fig 3E). We tried, but failed, to use the other *Sema-1a*-RNAi line, *UAS-Sema-1aRNAi*^*TRiP-V10*^ (which expresses *Sema-1a-*long double strand RNA (dsRNA) using the Valium 10 backbone vector [8]; BL29554) to verify the *Sema-1aRNAi*^*TRiP-V20*^-derived DA1 vPN morphological defects (data not shown and S1A Fig). However, this might not be a surprising result since it has been reported that Valium 10-based RNAi transgenes generally exhibit lower gene knock-down efficiency than Valium 20-based RNAi transgenes as described in the Bloomington RNAi stock webpage (http://flystocks.bio.indiana.edu/Browse/RNAi/RNAi_all.php) [8]. To confirm the authenticity of *Sema-1aRNAi*^*TRiP-V20*^-derived axonal and dendritic defects in the DA1 vPN, we have made the third line carrying another *Sema-1a-*RNAi transgene, *UAS-Sema-1aRNAi*^*sy*^, whose targeting sequence is distinct from the *UAS-Sema-1aRNAi*^*TRiP-V20*^ transgene. Intriguingly, the *Sema-1aRNAi*^*sy*^*-* expressing DA1 vPN did recapitulate the phenotypes of axonal mis-projection out of the LH and dendritic mis-targeting to the DA3 glomerulus (yellow triple-arrowhead and yellow triple-arrow Fig 3F). These *Sema-1a-*RNAi knock-down results again demonstrated the power of using R95B09-GAL4 as a manipulating reagent for the morphogenetic study of the DA1 vPN.

Besides *Dscam1* and *Sema-1a*, knock-down of *derailed* (*drl*), *Derailed 2* (*Drl-2*), *Eph receptor tyrosine kinase* (*Eph*), *Leukocyte-antigen-related-like* (*Lar*) and *Protein tyrosine phosphatase 4E* (*Ptp4E*) with RNAi transgenic lines (BL39002, BL25961, BL39066, BL34956, and BL 38369, respectively) resulted in a low penetration of dendritic phenotypes in the DA1 vPN (S1A–S1C Fig). LOF of *drl* (6%) and *Lar* (9%) exhibited aberrant dendritic phenotypes with a slightly higher percentage compared to the rest of LOF of three genes (~3%; S1A Fig). The aberrant dendritic phenotype observed in the *Lar*-RNAi knock-down samples included partial dendritic innervation in the DA1 glomerulus, dendritic mis-targeting to the DA4l glomerulus and excessive dendritic arborizations distributed in the VL1 glomerulus and the posterior ventrolateral AL (yellow double-arrows, yellow triple-arrows and yellow arrows, respectively, in S1B Fig). Intriguingly, LOF of *drl* and *Ptp4E* displayed a similar but less severe *Lar*-deficient dendritic defect (yellow double-arrows, yellow triple-arrows and yellow arrows, respectively, in S1C and S1D Fig). On the other hand, samples with LOF of *Drl-2* and *Eph* displayed dendritic shifting phenotypes from the DA1 glomerulus to ventroposterior and anteromiddle portions of the AL, respectively (yellow arrow, yellow double-arrows and yellow triple-arrows in S1E and S1F Fig). Interestingly, the dendrites of *Eph*-deficient DA1 vPN distributed roughly in three clusters along the anteromiddle portion of the AL, including the D, DA3 and DA4l glomeruli within the dorsal cluster, the VA1d and DC3 glomeruli within the middle cluster and the VA5 and VL1 glomeruli within the ventral cluster (yellow triple-arrows in S1F Fig). However, none of above phenotypes resulting from the loss of *Lar*, *Ptp4E*, *Drl-2* and *Eph* can be recapitulated by other independent RNAi transgenic lines (BL40938, BL60008, BL55893 and BL60006, respectively; S1A Fig), which kind of undermined the above finding for the roles of these genes in the DA1 vPN morphogenesis. Nonetheless, the RNAi knock-down results on *Dscam1* and *Sema-1a* together with the above results of the depiction of the developing DA1 vPN suggested that R95B09-GAL4 could serve as an ideal tool for future systematically studying the molecular and cellular basis of the DA1 vPN morphogenesis, which could lead to a better understanding of how vPNs are assembled into the olfactory system.

## Discussion

In the *Drosophila* olfactory neural circuit, neurites of OSNs, LNs and PNs are assembled together to form the primary olfactory center, the AL. Of the four PN groups, the development of adPNs and lPNs has been extensively explored, while the development of lvPNs and vPNs remains understudied [4]. In the current study, we investigate the molecular and cellular basis of the morphogenesis of a putative male-pheromone responding vPN, the DA1 vPN. At the cellular level, we depicted when and where stereotyped patterns of axonal branches and dendritic arborizations are established in the DA1 vPN. At the molecular level, we identified genes of cell surface molecules, including *Dscam1* and *Sema-1a*, which might be essential for forming the characteristic axonal and dendritic patterns of the DA1 vPN. By revealing the molecular and cellular mechanisms underlying the DA1 vPN morphogenesis, we should provide insights into how vPNs are assembled in the olfactory neural circuit.

In this study, we utilized an FLP-out intersection strategy to obtain a pure DA1 vPN labeling pattern, which allowed us to clearly depict the formation of axonal branches and dendritic arborizations of the DA1 vPN from early pupal stage to the adulthood. Intriguingly, both dendrites and axons of the DA1 vPN were initially accumulated at the ventral parts of the developing AL and LH, respectively, and its characteristic dorsal dendritic arborizations and axonal branches were not seen until the mid-early pupal stage (Fig 2). What is the molecular mechanism underlying the cellular processes for generating appropriate dendritic and axonal patterns of the DA1 vPN? From RNAi knock-down screen we found two molecules, Dscam1 and Sema-1a, which might play a crucial role in the DA1 vPN morphogenesis. Axonal aggregation and axonal mis-projection defects were observed in the DA1 vPN when *Dscam1* and *Sema-1a* were knocked down, which were the similar axonal phenotypes that exhibited in other types of neurons (Fig 3A–3C) [14, 15]. In contrast, a novel phenotype of mis-targeted dendrites to the DA3 glomerulus was observed when *Sema-1a* was deficient in the DA1 vPN (Fig 3A and 3C). Previous reports have demonstrated that Sema-1a operates as a receptor for PNs to regulate the process of initial dendritic targeting via the interaction with secreted semaphorins, Sema-2a and Sema-2b, at the early pupal stage [15, 16]. This initial dendritic targeting process was mediated via two opposite gradients of Sema-1a and Sema-2a/2b in the developing AL: the dorsolateral (DL) expression of Sema-1a in dendrites of PNs and the ventromedial (VM) expression of Sema-2a/2b in axons of degenerating larval OSNs [15, 16]. The dendrites of DL1 adPNs and DA1 lPNs tended to make a DL-to-VM shift when *Sema-1a* was removed from these PNs or *Sema-2a/2b* was deficient from the degenerating larval OSNs [15, 16]. In our current study, however, we found that the dendrites of the DA1 vPN were specifically mis-targeted to the DA3 glomerulus when *Sema-1a* was deficient (Fig 3A and 3C). It is interesting, although perplexing at the same time, that the same molecule, i.e., Sema-1a, plays distinct roles in forming appropriate dendritic patterns for different types of PNs. Through loss-of-function and rescue studies, we have demonstrated that Sema-1a plays a novel role in the prevention of aberrant dendritic invasion specifically into the DA3 glomerulus from surrounding adPNs (D, DA4m, DA4l, DC3 and VA1d adPNs) and vPNs (DA1 and diffuse vPNs) but not lPNs (DA1 and DL3 lPNs), which is different from the previously described function of Sema-1a for its graded expression in guiding PN initial dendritic targeting within the AL (*Shen et al*., manuscript in preparation).

Unlike most of adPNs and lPNs with uni-glomerular dendritic patterns, dendrites of many types of vPNs innervate multiple glomeruli in the AL [17, 18]. Interestingly, animals with LOF of *Lar*, *drl*, *Drl-2*, *Eph* and *Ptp4E*, despite of occurring in a low frequency, all displayed a tendency to switch their dendritic innervation from single DA1 glomerulus into multiple glomeruli (Fig 3A and 3D–3H). This observation raises a reasonable speculation in which the initial ventral localization of axons and dendrites of the DA1 vPN represents a default state for the development of vPNs and perturbation of the gene expression of cell surface molecules like *Lar*, *drl*, *Drl-2*, *Eph* and *Ptp4E* could lead to dendritic arborizations of vPNs from uni-glomerulus into multiple glomeruli in the AL. Nonetheless, future investigation is required to validate whether the dendritic morphogenesis of the DA1 vPN and various types of vPNs might be sculpted by various combinations of cell surface molecules. Revealing the molecular mechanism underlying the regulation of vPN morphogenesis should promise a comprehensive understanding of how the complex olfactory system is formed in the brain to decode odorant information from the external world.

## Materials and Methods

### Making the fly stock carrying a RNAi transgene for knock-down of *Sema-1a*

Standard molecular biological techniques were used to generate *UAS-Sema-1aRNAi*^*sy*^ which encodes microRNA (miRNA) carrying the unique *Sema-1a* sequence, *agagcaaggatcaggaaataat*, for knocking down the expression of *Sema-1a*. The design of miRNA for knock-down of *Sema-1a* followed the strategy described previously [19]. The cDNA containing the miRNA backbone with tandem repeats of *Sema-1a* target sequence was subsequently cloned into pJFRC7-20XUAS-IVS-mCD8::GFP in Xho 1 and Xba 1 sites [20]. The cDNA construct of *UAS-Sema-1aRNAi*^*sy*^ was injected into the fly stock carrying an attP docking site (VK00033; e.g., BL9750) to generate the transgenic fly via the service provided by Rainbow Transgenic Flies, Inc.

### Fly strains

The fly strains used in this study were as follows: (1) *R95B09-GAL4* (BL47267); (2) *UAS-mCD8*::*GFP* [21]; (3) *GH146-FLP* [10]; (4) *UAS-FRT<stop<FRT-myr-GFP* [20]; (5–7) *UAS-Dscam1RNAi*^*17*.*1i*^, *UAS-Dscam1RNAi*^*17*.*2i*^ and *UAS-Dscam1 RNAi*^*18i*^*;* [13]; (8) *UAS-Sema-1aRNAi*^*sy*^; (9–47) *UAS-RNAi*^*TRiP*^ stocks from the TRiP collection (see the S1 table for the list of these *UAS-RNAi*^*TRiP*^ stocks).

### Fly brain preparation for molecular and cellular studies of the DA1 vPN morphogenesis

Dissection, immunostaining, and mounting of fly brains were performed as described in a standard protocol [21]. Primary antibodies used in this study included rabbit antibody against GFP (1:800, Invitrogen), rat monoclonal antibody against DN-cadherin (DN-Ex#8, 1:100, DSHB) and mouse monoclonal antibodies against Bruchpilot (nc82, 1:100, DSHB), Fasciclin II (1D4, 1:50, DSHB) and Neurotactin (BP106, 1:50, DSHB). Secondary antibodies conjugated to different fluorophores (Alexa 488, 546, and 647 (Invitrogen)) were used at a 1:800 dilution in this study. Immunofluorescent images were collected by Zeiss LSM 700 or 780 confocal microscopy and projected using LSM browser.

## Supporting Information

S1 Fig A pilot RNAi screen to identify molecules potentially important for the DA1 vPN morphogenesis. The result of the pilot RNAi knock-down screen for the DA1 vPN morphogenesis was summarized in the panel A. Images of DA1 vPN phenotypes with gene perturbation were shown in green: *Lar* (B), *drl* (C), *Ptp4E* (D), *Drl-2* (E) and *Eph* (F). Brain neuropiles were stained with an antibody against Brp (shown in blue in B-F). Characteristic dorsal and ventral axonal branches were indicated by arrowheads and double-arrowheads in B-F. (A) Percentage of DA1 vPN phenotypes in the bar graph was used to show the effect of RNAi knock-down of 35 genes. The vertical axis indicated the RNAi lines used for knocking down 35 genes with their Bloomington stock number (BL) or original sources and with their examined sample sizes (n). (B-D) Similar DA1 vPN dendritic defects were observed in animals of LOF of *Lar*, *drl* and *Ptp4E*: lacking a fully dendritic innervation in the DA1 glomerulus (yellow double-arrows), mis-targeting dendrites to the DA4l glomerulus (yellow triple-arrows) and arborizing excessive dendrites in the VL1 glomerulus and the posterior ventrolateral AL (yellow arrows). (E and F) DA1 vPN dendritic shifting phenotypes were observed in *Drl-2* and *Eph* RNAi knock-down animals, in which the dendritic innervation was absent from the DA1 glomerulus (yellow double-arrows) and found to distribute at ventroposterior and anteromiddle portions of the AL, respectively (yellow arrows, yellow double-arrows and yellow triple-arrows). The dendrites of *Eph*-deficient DA1 vPN were found as three clusters within the AL: (1) D, DA3 and DA4l glomeruli, (2) VA1d and DC3 glomeruli and (3) VA5 and VL1 glomeruli (yellow triple-arrows). Scale bar: 10 μm.
(TIF)

S1 Table *UAS-RNAi*^*TRiP*^ fly stocks used in the current study. The *UAS-RNAi*^*TRiP*^ fly stocks used in this study that were available from Bloomington *Drosophila* stock center were summarized with their names of targeting genes, stock numbers (BL#), Valium vectors for constructing RNAi transgenes, attP integration sites for generating RNAi transgenic fly stocks and the targeting sequence information of RNAi transgenes.
(DOCX)

S2 Table Genotypes of flies in the experiments shown in the indicated figure panels. Genotypes of flies in the Figs 1–3 and S1 Fig were summarized in this supplemental table.
(DOCX)
