# Supplementary figures and images for: Morphogenetic Studies of the Drosophila DA1 Ventral Olfactory Projection Neuron
Source: PLoS One. 2016 May 10;11(5):e0155384. doi: 10.1371/journal.pone.0155384 (PMC4862648; doi:10.1371/journal.pone.0155384)

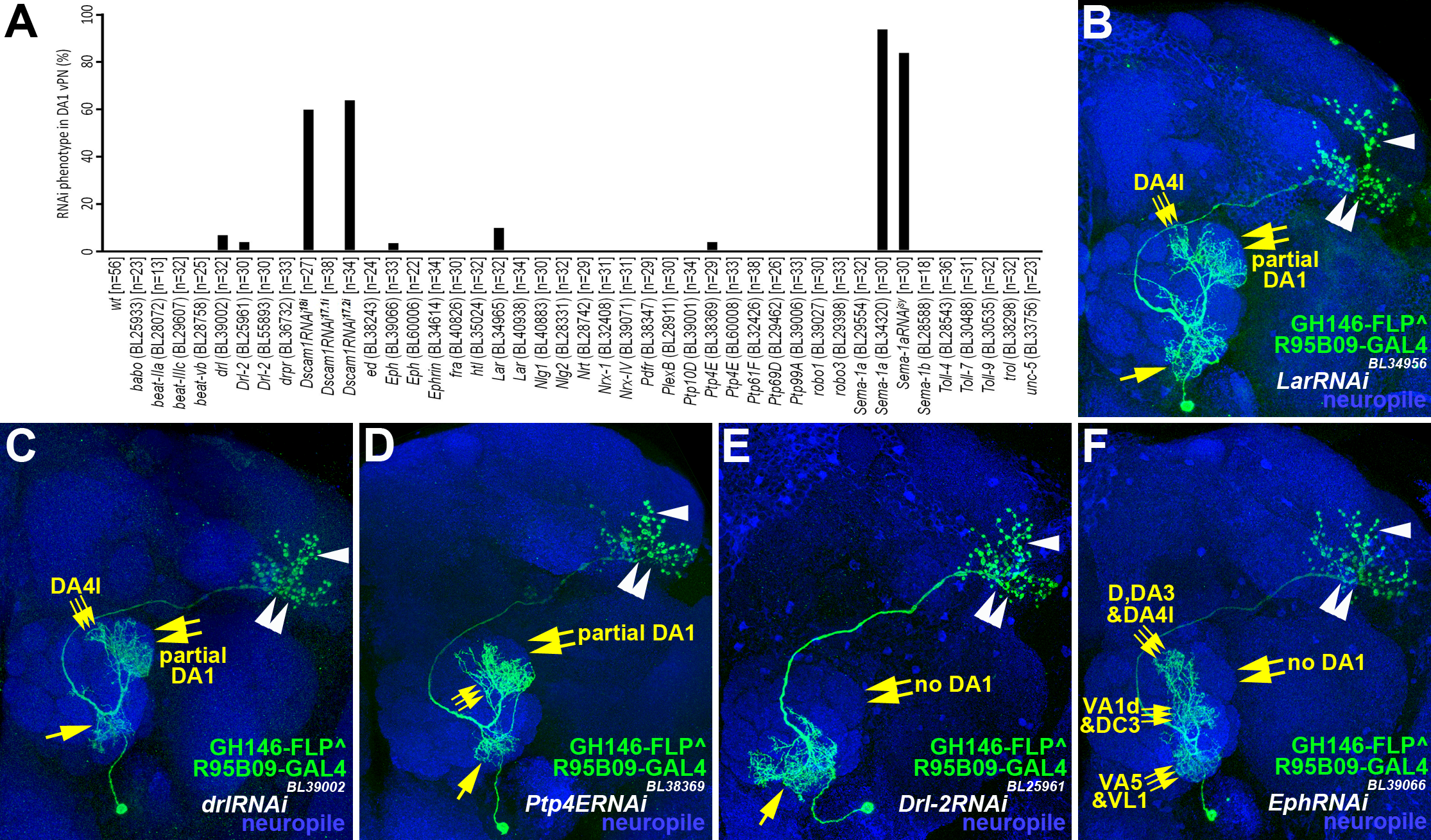

Supplement: S1 Fig — The result of the pilot RNAi knock-down screen for the DA1 vPN morphogenesis was summarized in the panel A. Images of DA1 vPN phenotypes with gene perturbation were shown in green: Lar (B), drl (C), Ptp4E (D), Drl-2 (E) and Eph (F). Brain neuropiles were stained with an antibody against Brp (shown in blue in B-F). Characteristic dorsal and ventral axonal branches were indicated by arrowheads and double-arrowheads in B-F. (A) Percentage of DA1 vPN phenotypes in the bar graph was used to show the effect of RNAi knock-down of 35 genes. The vertical axis indicated the RNAi lines used for knocking down 35 genes with their Bloomington stock number (BL) or original sources and with their examined sample sizes (n). (B-D) Similar DA1 vPN dendritic defects were observed in animals of LOF of Lar, drl and Ptp4E: lacking a fully dendritic innervation in the DA1 glomerulus (yellow double-arrows), mis-targeting dendrites to the DA4l glomerulus (yellow triple-arrows) and arborizing excessive dendrites in the VL1 glomerulus and the posterior ventrolateral AL (yellow arrows). (E and F) DA1 vPN dendritic shifting phenotypes were observed in Drl-2 and Eph RNAi knock-down animals, in which the dendritic innervation was absent from the DA1 glomerulus (yellow double-arrows) and found to distribute at ventroposterior and anteromiddle portions of the AL, respectively (yellow arrows, yellow double-arrows and yellow triple-arrows). The dendrites of Eph-deficient DA1 vPN were found as three clusters within the AL: (1) D, DA3 and DA4l glomeruli, (2) VA1d and DC3 glomeruli and (3) VA5 and VL1 glomeruli (yellow triple-arrows). Scale bar: 10 μm. (TIF) [file pone.0155384.s001.tif]
