# Supplementary material for: Morphogenetic Studies of the Drosophila DA1 Ventral Olfactory Projection Neuron
Source: PLoS One. 2016 May 10;11(5):e0155384. doi: 10.1371/journal.pone.0155384 (PMC4862648; doi:10.1371/journal.pone.0155384)
Supplement: S1 Table — The UAS-RNAiTRiP fly stocks used in this study that were available from Bloomington Drosophila stock center were summarized with their names of targeting genes, stock numbers (BL#), Valium vectors for constructing RNAi transgenes, attP integration sites for generating RNAi transgenic fly stocks and the targeting sequence information of RNAi transgenes. (DOCX) [file pone.0155384.s002.docx]

**S1 Table.** *UAS-RNAi^TRiP^* fly stocks used in the current study.

| **Gene** | **BL#** | **Valium** | **attP site** | **Info. for the targeting sequence** |
| --- | --- | --- | --- | --- |
| *babo* | 25933 | 10 | attP2 | 415bp dsRNA |
| *beat-IIa* | 28072 | 10 | attP2 | 429bp dsRNA |
| *beat-IIIc* | 29607 | 10 | attP2 | 448bp dsRNA |
| *beat-vb* | 28758 | 10 | attP2 | 417bp dsRNA |
| *drl* | 39002 | 20 | attP | 21bp shRNA; *caagtgtgtgtttatgtctaa* |
| *Drl-2* | 25961 | 10 | attP2 | 441bp dsRNA |
| *Drl-2* | 55893 | 20 | attP2 | 21bp shRNA; *tcggagttcagtgctgatata* |
| *drpr* | 36732 | 20 | attP2 | 21bp shRNA; *tagctattcgatcaactacaa* |
| *ed* | 38243 | 20 | attP40 | 21bp shRNA; *taggattagagttccgatcta* |
| *Eph* | 39066 | 20 | attP40 | 21bp shRNA; *acgttgttgtttgttgatcaa* |
| *Eph* | 60006 | 20 | attP40 | 21bp shRNA; *tacaagatcaagtgtaatata* |
| *Ephrin* | 34614 | 20 | attP2 | 21bp shRNA; *cagattcatgacagttgtcaa* |
| *fra* | 40826 | 20 | attP2 | 21bp shRNA; *ctggtcgatgtctgtgttaaa* |
| *htl* | 35024 | 20 | attP2 | 21bp shRNA; *tcggttgtatttgctgttgta* |
| *Lar* | 34965 | 20 | attP2 | 21bp shRNA; *atggagaaggatgtcaatcaa* |
| *Lar* | 40938 | 20 | attP40 | 21bp shRNA; *cagatggtcgacaatagcgaa* |
| *Nlg1* | 40883 | 20 | attP40 | 21bp shRNA; *accgacgaaggttccagcgaa* |
| *Nlg2* | 28331 | 10 | attP2 | 408bp dsRNA |
| *Nrt* | 28742 | 10 | attP2 | 439bp dsRNA |
| *Nrx-1* | 32408 | 20 | attP2 | 21bp shRNA; *cggaacgaaggtcaacgaca* |
| *Nrx-IV* | 39071 | 20 | attP40 | 21bp shRNA; *tagcatgtgtatatagatata* |
| *Pdfr* | 38347 | 20 | attP40 | 21bp shRNA; *accaatagacatttaatttaa* |
| *PlexB* | 28911 | 10 | attP2 | 402bp dsRNA |
| *Ptp10D* | 39001 | 20 | attP2 | 21bp shRNA; *tcggtgtgatcaagaatctaa* |
| *Ptp4E* | 38369 | 20 | attP2 | 21bp shRNA; *cacgtacgaagtggtggtcaa* |
| *PtP4E* | 60008 | 20 | attP40 | 21bp shRNA; *cagacagtgcatacgaatcta* |
| *Ptp61F* | 32426 | 20 | attP2 | 21bp shRNA; *cagagcagttctcatgctcaa* |
| *Ptp69D* | 29462 | 10 | attP2 | 550bp dsRNA |
| *Ptp99A* | 39006 | 20 | attP2 | 21bp shRNA; *cagcagcgacagatgctcaaa* |
| *robo1* | 39027 | 20 | attP40 | 21bp shRNA; *acgaggaatgtttgttagaaa* |
| *robo3* | 29398 | 10 | attP2 | 576bp dsRNA |
| *Sema-1a* | 29554 | 10 | attP2 | 422bp dsRNA |
| *Sema-1a* | 34320 | 20 | attP2 | 21bp shRNA; *tcgatcaatgtcagcaaacaa* |
| *Sema-1b* | 28588 | 10 | attP2 | 487bp dsRNA |
| *Toll-4* | 28543 | 10 | attP2 | 479bp dsRNA |
| *Toll-7* | 30488 | 10 | attP2 | 255bp dsRNA |
| *Toll-9* | 30535 | 10 | attP2 | 255bp dsRNA |
| *trol* | 38298 | 20 | attP40 | 21bp shRNA; *caggatctgctcacactgcta* |
| *unc-5* | 33756 | 20 | attP2 | 21bp shRNA; *caggaagaatgtagcgtttaa* |
