# Supplementary material for: Morphogenetic Studies of the Drosophila DA1 Ventral Olfactory Projection Neuron
Source: PLoS One. 2016 May 10;11(5):e0155384. doi: 10.1371/journal.pone.0155384 (PMC4862648; doi:10.1371/journal.pone.0155384)
Supplement: S2 Table — Genotypes of flies in the Figs 1–3 and S1 Fig were summarized in this supplemental table. (DOCX) [file pone.0155384.s003.docx]

**S2 Table. Genotypes of flies in the experiments shown in the indicated figure panels.**

| **Fig** | **Genotype** |
| --- | --- |
| 1 | *w;UAS-mCD8::GFP;R95B09-GAL4;+* |
| 2B & 2E-2H | *w;GH146-FLP/+;R95B09-GAL4(attP2),UAS-FRT<stop<FRT-myr-GFP/+;+* |
| 2C&2D | *w;GH146-FLP;R95B09-GAL4,UAS-FRT<stop<FRT-myr-GFP;+* |
| 3A | *w;GH146-FLP/+;R95B09-GAL4,UAS-FRT<stop<FRT-myr-GFP/UAS-RNAi;+* |
| 3B | *w;GH146-FLP/UAS-Dscam1RNAi^18i^;R95B09-GAL4,UAS-FRT<stop<FRT-myr-GFP/+;+* |
| 3C | *w;GH146-FLP/UAS-Dscam1RNAi^17.1i^;R95B09-GAL4,UAS-FRT<stop<FRT-myr-GFP/+;+* |
| 3D | *w;GH146-FLP/UAS-Dscam1RNAi^17.2i^;R95B09-GAL4,UAS-FRT<stop<FRT-myr-GFP/+;+* |
| 3E | *w;GH146-FLP/+;R95B09-GAL4,UAS-FRT<stop<FRT-myr-GFP/ UAS-Sema-1aRNAi^TRiP-V20^;+* |
| 3F | *w;GH146-FLP/+;R95B09-GAL4,UAS-FRT<stop<FRT-myr-GFP/ UAS-Sema-1aRNAi^sy^;+* |
| S1B-S1F | *w;GH146-FLP/UAS-RNAi^TRiP^;R95B09-GAL4,UAS-FRT<stop<FRT-myr-GFP/+;+* or  *w;GH146-FLP/+;R95B09-GAL4,UAS-FRT<stop<FRT-myr-GFP/UAS-RNAi^TRiP^;+* |
